# Menstrual hygiene management in schools: midway progress update on the “MHM in Ten” 2014–2024 global agenda

**DOI:** 10.1186/s12961-020-00669-8

**Published:** 2021-01-02

**Authors:** Marni Sommer, Bethany A. Caruso, Belen Torondel, Elodie C. Warren, Brooke Yamakoshi, Jackie Haver, Jeanne Long, Thérèse Mahon, Ella Nalinponguit, Neville Okwaro, Penelope A. Phillips-Howard

**Affiliations:** 1 Mailman School of Public Health, Columbia University, New York, NY United States of America; 2 Rollins School of Public Health, Emory University, Atlanta, GA United States of America; 3 London School of Hygiene and Tropical Medicine, London, United Kingdom; 4 UNICEF, New York, NY United States of America; 5 Save the Children US, Washington, DC United States of America; 6 WaterAid, London, United Kingdom; 7 Department of Education, Manila, The Philippines; 8 Ministry of Health, Nairobi, Kenya; 9 Liverpool School of Tropical Medicine, Liverpool, United Kingdom

**Keywords:** Adolescent health, Menstruation, Equity, Education, Girl’s education, Policy

## Abstract

Progress has been made in recent years to bring attention to the challenges faced by school-aged girls around managing menstruation in educational settings that lack adequate physical environments and social support in low- and middle-income countries. To enable more synergistic and sustained progress on addressing menstruation-related needs while in school, an effort was undertaken in 2014 to map out a vision, priorities, and a ten-year agenda for transforming girls’ experiences, referred to as Menstrual Hygiene Management in Ten (MHM in Ten). The overarching vision is that girls have the information, support, and enabling school environment for managing menstruation with dignity, safety and comfort by 2024. This requires improved research evidence and translation for impactful national level policies. As 2019 marked the midway point, we assessed progress made on the five key priorities, and remaining work to be done, through global outreach to the growing network of academics, non-governmental organizations, advocates, social entrepreneurs, United Nations agencies, donors, and national governments. This paper delineates the key insights to inform and support the growing MHM commitment globally to maximize progress to reach our vision by 2024. Corresponding to the five priorities, we found that (priority 1) the evidence base for MHM in schools has strengthened considerably, (priority 2) global guidelines for MHM in schools have yet to be created, and (priority 3) numerous evidence-based advocacy platforms have emerged to support MHM efforts. We also identified (priority 4) a growing engagement, responsibility, and ownership of MHM in schools among governments globally, and that although MHM is beginning to be integrated into country-level education systems (priority 5), resources are lacking. Overall, progress is being made against identified priorities. We provide recommendations for advancing the MHM in Ten agenda. This includes continued building of the evidence, and expanding the number of countries with national level policies and the requisite funding and capacity to truly transform schools for all students and teachers who menstruate.

## Background

Over the last decade, evidence has accrued from around the world on the many barriers faced by schoolgirls to safe, hygienic, and dignified menstruation [1]. Challenges include limited or nonexistent information prior to menstrual onset [2]; inadequate health education about menstruation and puberty [3]; a lack of social support from teachers and peers for managing menses in school, and from families [4]; and insufficient access to water, sanitation, hygienic materials and disposal infrastructure. These barriers contribute to gender discriminatory physical school environments [5, 6] and pervasive menstruation-related stigma, enabling behavioral restrictions and feelings of shame, stress, and taboo [7]. Menstruation-related barriers are also associated with girls’ education [8–10]. This includes, for example, difficulty participating and engaging in the classroom, and thus achieving their potential, along with missed hours or days of school, and anxiety around menstrual accidents [11, 12]. Researchers have also identified associations with transactional sex in exchange for sanitary pads [13], increased vulnerability to pregnancy or child marriage, with subsequent school dropout or expulsion [14–16]; and bullying or teasing about menstruation by school boys [17]. These gendered educational realities may lead to negative reproductive and psychosocial outcomes, and diminished future economic opportunities. In turn, this may reinforce gender inequalities globally. Assuring the ability to manage menstruation safely and with dignity is essential to meeting the Sustainable Development Goals (SDGs) for gender equality, good health, quality education, and sustainable water and sanitation for all; and related human rights. An essential aspect of addressing this issue are evidence-informed national level policies, and the resources to support their implementation.

As interest grew globally on the issue of menstruation in schools, an effort was undertaken to develop a ten-year agenda (2014–2024) with five corresponding priorities that address the intersection of girls’ education, health, gender and menstruation (Box 1). This endeavor incorporated inputs from global experts working on the issue of menstrual hygiene management (MHM), including academics, United Nations (UN) agencies, non-governmental (NGO) and advocacy organizations, private sector, social entrepreneurs, and national governments. The term MHM is used given it was the predominant term at the time referring to the components of managing menstruation with dignity and comfort, as defined by the Joint Monitoring Programme in 2012. The overarching aim was to collectively achieve, by 2024, a “MHM in Ten” vision [18]:

MHM in Ten Vision: Girls in 2024 around the world are knowledgeable and comfortable with their menstruation, and able to manage their menses in school in a comfortable, safe and dignified way.

The articulation of a “MHM in Ten” agenda and key priorities was a call to action to identify synergies and encourage and mobilize coordinated—rather than duplicative—efforts to address the MHM needs of schoolgirls through providing a roadmap for stakeholder engagement at global, national, and subnational levels, in the public and private sectors, and among development and humanitarian response actors [6].

Numerous organizations and institutions have incorporated the MHM in Ten vision and priorities into their menstruation-related planning and programmes, and the call to action has been cited over 108 times [18]. UNICEF continues to promote the importance of synergistic action in making progress on the agenda through requesting that during its annual virtual conference on MHM in schools indicate which priority (or priorities) the showcased work is advancing. Globally the evidence for action continues to grow, and a select number of national or sub-national level menstrual policies have been enacted. Five years have passed since the launching of the agenda, prompting a need to examine if and how progress is being achieved midway between the generation of the priorities and the ten-year endpoint. The aim of this paper is to present the findings from a narrative review conducted of research, programmes, policies, and initiatives focused on menstruation in schools across low- and middle-income countries to assess progress and gaps across the five priority areas identified, and to offer policy-oriented recommendations for global, regional, national, and subnational stakeholders. The insights from this review are intended for all actors working on MHM in schools to inform future effort, engagement, and investment required to achieve the collective MHM in Ten vision by 2024.

To review progress, we conducted a global desk review from April to June 2019. First, we identified 119 studies, programme reports, policies, systematic reviews, guidance documents and other documentation by searching a range of databases (Google, Google Scholar, PubMed, internal university database [CLIO] which catalogues over seven million articles, books, and archival content). Search terms included multiple combinations of the terms “advocacy”, “cost-effectiveness”, “data”, “effectiveness”, “evaluation”, “feasibility”, “gender”, “girls”, “global”, “government”, “guidance”, “guideline”, “impact”, “implementation”, “inclusive”, “indicator”, “international”, “menstrual health”, “menstrual hygiene management (MHM)”, “menstruation”, “national”, “policy”, “report”, “review”, “school(s)”, “systematic review”, “tool(kit)”, “trial”, “water, sanitation, and hygiene (WASH)”. These terms were also combined with the names of relevant organizations, networks, countries, regions, or international institutions to enhance relevance of results. Second, we contacted a total of 115 experts across sectors, including academics, NGOs, UN agencies, donors, governments, social entrepreneurs and advocates working in the MHM arena. Approximately 40% of the responses came from experts representing low-and middle-income countries, including government agencies, international agency country offices, and local NGOs. We began with all of the multi-sector participants of the initial and subsequent two annual follow-up MHM in Ten meetings held in 2014, 2015 and 2016 as well as those who presented at the UNICEF annual virtual conference on MHM in Schools. Electronic communications sent to all experts asked for: (1) updates and examples of progress being made to meet the five priorities; and (2) suggestions of additional organizations or experts to contact. Next, two members of the research team extracted information relevant to the five identified priorities from the documents identified from our search and the updates from experts. For the latter, we utilized an analysis approach developed in advance that incorporated key aspects of the priorities which we hoped to identify in relation to progress being made or not. These included, for example, examples of an expanding research base and general trends, the development of tools, the global state of the evidence, the inclusivity of research and programming, and other relevant categories that would indicate advancement or lack thereof. Finally, we selected examples across the priorities that illustrated progress being made or gaps where minimal progress has been achieved.

Box 1: Five priorities of the MHM in Ten Agenda *Priority* *1*: Build a strong cross-sectoral evidence base for MHM in schools for prioritization of policies, resource allocation, and programming at scale.

*Priority* *2*: Develop and disseminate global guidelines for MHM in schools with minimum standards, indicators, and illustrative strategies for adaptation, adoption, and implementation at national and subnational levels.

*Priority* *3*: Advance MHM in schools activities through a comprehensive evidence-based advocacy platform that generates policies, funding, and action across sectors and at all levels of government.

*Priority* *4*: Allocate responsibility to designated government entities for the provision of MHM in school, including adequate budget and monitoring and evaluation (M&E); and the reporting to global channels and constituents.

*Priority* *5*: Integrate MHM and the capacity and resources to deliver inclusive MHM into the education system.

## Update of progess since 2014

In the following sections we discuss each priority, detail known initiatives, and briefly note where further progress is urgently needed, particularly in the realm of evidence and policy. We acknowledge that all efforts globally are not captured, but have aimed to provide illustrative examples across sectors. The summaries below are intended to assess collective progress made by the global community working on the issue of menstruation across the articulated priorities. We do not intend to imply that that the MHM in Ten agenda is responsible for the initiatives noted. We have included in the Tables 1, 2, 3, 4, 5 exemplars of progress and gaps across each priority, along with research, policy and related recommendations for advancing the agenda. In addition, we chose to include a select number of additional achievements across the MHM global community that while not specifically school-based, represent assessments or actions which contribute to national policy discussions and highlight population-level inequities, both of which are necessary to inform school menstrual health and hygiene policies and programmes.

*Priority* *1:* Build a strong cross-sectoral evidence base for MHM in schools for prioritization of policies, resource allocation, and programming at scale.

The evidence base for MHM in schools has strengthened considerably since 2014. A key outcome of the first MHM in Ten meeting was the publication of research priorities for the 10-year agenda that summarized the evidence to date, articulated the gaps, recommended methodologies for future learning, and included a call for standardized measures within and across key sectors [19]. Progress has been made against the research needs that were identified. Overall, support from global funding bodies and research councils has increased, facilitating research beyond qualitative and descriptive studies [20] to include cross-sectional studies in Bangladesh [21], pilot intervention trials in Kenya, Uganda and the Gambia including varying combinations of WASH, sexual and reproductive health (SRH) and education [23], intervention studies in Bangladesh and Uganda [24, 25]; full intervention trials in Kenya, and systematic reviews with meta-analyzes analyzing MHM-related evidence and measures and outcomes being utilized [1, 9, 10, 26–29]. In Kenya and the Philippines (see Boxes 2 and 3), evidence has fed directly into national policy and monitoring efforts influencing both countries’ education systems. These findings suggest that while important progress has been made to advance the evidence base, there is now a need for implementation science research to learn from these and other initiatives to understand how successful interventions, policies and programmes are delivered and scaled. In particular, the recommendations for advancing the agenda (see Table 1) articulate a range of empirical evidence needed, with topical and methodological areas described. This will be fundamental to informing advocacy efforts, along with and national and sub-national level policy.

**Table 1:** Progress and gaps identified related to priority 1

Priority 1: Build a strong cross-sectoral evidence base for MHM in schools for prioritization of policies, resource allocation, and programming at scale	
Progress	
	1. *New* *indicators* *and* *tools* *in* *use* *and* *under* *development* *for* *intervention* *research* *and* *monitoring* *in* *relation* *to* *MHM* *and* *WASH* *in* *schools*
New indicators and tools	a. Indicators and data collection efforts for monitoring at global levels:
	Joint Monitoring Programme (JMP) to track progress related to SDG targets 6.1, 6.2 and 4a
	UNICEF’s Multiple Cluster Indictor Survey (MICS) added questions on menstruation
	Inclusion of MHM (WASH and menarche) questions in the Demographic and Health Survey
	b. Indicators and data collection efforts for research and monitoring at regional and national levels:
	Bangladesh National Hygiene Baseline Survey
	WaterAid and UNICEF’s analysis of MHM in schools in South Asia
	Grand Challenges Canada’s comparative analysis of MHM innovative enterprises
	Performance Monitoring and Accountability (PMA) 2020 (Johns Hopkins) produced MHM country-specific reports of household level data on women over age 15 in select countries
	2. *New* *tools* *being* *developed* *and* *tested* *(reaching* *beyond* *school-based* *activities)*
	Menstrual self-efficacy (Johns Hopkins Bloomberg School of Public Health and International Center Diarrheal Disease Bangladesh (ICDDRB)); Menstruation ENgagement, Self-efficacy, and Stress (MENSES) (Save the Children)
	Global monitoring experts in health (sexual and reproductive / psychosocial), gender, education, and WASH identified outcome/impact measures for addressing menstrual health and hygiene among girls globally (Columbia University; WSSCC)
Expansion of evidence	*More* *expansive* *inquiries* *and* *collaborative* *efforts* *between* *academic* *institutions,* *intergovernmental* *organizations,* *global* *and* *local* *nonprofit* *organizations,* *and* *the* *private* *sector*
	Research in Kenya, Uganda, Zambia, Gambia, Bangladesh, Bolivia, Bhutan, Fiji, Mongolia, and Pakistan among others (Muthengi, Farris, and Austrian 2017; Hennegan et al. n.d.; Department of School Education of Bhutan and United Nations Children Fund 2018; Johnson et al. 2016; Miiro et al. 2018; Chinyama et al. 2019; Alam et al. 2017; WaterAid Australia 2017; Mumtaz, Sommer, and Bhatti 2016) on the MHM-related challenges faced by girls and female teachers, toilet needs within religious backgrounds
	Feasibility studies, impact evaluations, effectiveness and cost-effectiveness trials (Zulaika et al. 2019; Sommer 2010; Hennegan and Montgomery 2016; Alexander et al. 2015; Miiro et al. 2018; Sommer et al. 2018; Emdadul Haque et al. 2014; Muthengi, Farris, and Austrian 2017)
	Research analyzing the impact of the MHM and education policy landscape (Sommer et al. 2017)
	MHM among people with disabilities (Wilbur et al. 2019), boys and men’s perceptions of MHM for schoolgirls (Mason et al. 2017), MHM in emergency contexts (Sommer et al. 2018)
Cross-sector Collaboration	*New* *efforts* *to* *mobilize* *research* *networks* *and* *synergies,* *including*
	The Global Challenges Research Fund (GCRF) seed grant among researchers in Kenya, Uganda, Tanzania, and Zimbabwe to strengthen MHM research capacity with a particular focus on schools
	MHM in Emergencies toolkit (27 co-publishing humanitarian response organizations), with inclusion of MHM in education sector response (Sommer et al. 2018)
Recommendations to advance the agenda	
Longitudinal research on the consequences of poor MHM, and the synergistic impact of combined MHM interventions for schoolgirls, including the long-term effect of such interventions	
More inclusive studies, involving men and boys, parents, teachers, and marginalized groups such as girls with disabilities and transgendered youth	
Systematic reviews evaluating MHM interventions and outcomes across differing geographies, cultures, population groups	
Financial modeling for and cost-effectiveness of MHM programmes and policies in schools to promote uptake of evidence-based research, dissemination, cross-sectoral engagement, and implementation	
‘Natural experiments’ to understand impact of policies and funding for MHM programmes in schools, including those that provide menstrual products to girls in high, middle, and low- income countries	
Implementation research to understand how programmes and policies are delivered, adapted, and scaled	

This table comprehensively outlines specific priority 1 progress and gaps to date

*Priority* *2:* Develop and disseminate global guidelines for MHM in schools with minimum standards, indicators, and illustrative strategies for adaptation, adoption, and implementation at national and subnational levels.

Although there does not exist broad consensus for one set of global guidelines, global and national level guidance for MHM in schools has been developed and disseminated (see Table 2). Country-specific examples include the Kenyan government’s design of guidelines on MHM strategy, policy, and a manual for training teachers, utilizing a multi-sector approach, and spearheaded by the Ministry of Health’s WASH group (see Box 2). Another country with existing guidelines is the Philippines government, which provides a useful example of a country that has integrated MHM indicators into their Education Monitoring Information System (EMIS). Early reports suggest that school-level MHM monitoring incentives improve compliance with standards, including the availability of emergency menstrual products in schools and the provision of adequate WASH facilities and MHM education (see Box 3).

**Table 2:** Progress and gaps related to priority 2

Priority 2: Develop and disseminate global guidelines for MHM in schools with minimum standards, indicators, and illustrative strategies for adaptation, adoption, and implementation at national and subnational levels	
Progress	
Global level guidelines	Menstruation and MHM incorporated within the 2014 UNESCO *Puberty* *Education* *&* *Menstrual* *Hygiene* *Management* guidelines, and the 2018 UNESCO *International* *Technical* *Guidance* *on* *Sexuality* *Education:* *An* *Evidence-Informed* *Approach* (UNESCO; UNAIDS; UNFPA; UNICEF; UN Women; WHO 2018; United Nations Educational Scientific and Cultural Organization 2014)
	UNICEF prioritized dignified menstrual health and hygiene in its *Gender* *Action* *Plan* *for* *2018–2021,* although this effort goes beyond girls in school (UNICEF n.d.)
	Save the Children’s 2016 *Menstrual* *Hygiene* *Management* *Operational* *Guidelines* provide comprehensive global guidelines for situation analysis, programme design, and monitoring and evaluation of ongoing MHM interventions in schools (Haver and Long 2016)
	School-specific evidence-based guidelines have emerged from governments (e.g. India, Zambia, Kenya), NGOs and international organizations with specific recommendations for WASH and MHM. Examples include USAID’s 2015 Schools Promoting Learning Achievement through Sanitation and Hygiene (SPLASH) *Menstrual* *Hygiene* *Management* *Toolkit* for Zambia, and World Vision India’s 2017 guidelines on *Making* *Schools* *Gender-Friendly* (SPLASH 2015; World Vision India 2017)
Country level guidelines	The Government of Nepal is rolling out the *National* *WASH* *in* *Schools* *Procedure* (2018) that incorporates standards for MHM education, materials and WASH facilities
*Recommendations* *to* *advance* *the* *agenda*	
Include MHM monitoring systems within global monitoring frameworks, and seek to capture fidelity and impact of MHM interventions in schools	
Increase political interest in designing and monitoring MHM enabling infrastructure in schools (gender sensitive facilities, cleaning and maintenance) aligned with SDGs	
Develop improved guidelines and indicators for use at varying levels, including schools, from national monitoring to programme implementation	
Documentation of how existing guidelines are disseminated, adapted, and integrated into local contexts, with particular focus on implementation in relation to education	

This table comprehensively outlines specific priority 2 progress and gaps to date

Multiple efforts have fed into the development and provision of global level guidance. Since 2014, global guidance has emerged from multi-sectoral partnerships specific to the WASH and education sectors. Save the Children (2016) and UNICEF (2019) developed guidance on Menstrual Health and Hygiene, which encompasses the project cycle through design, delivery and evaluation UNICEF outlines a package of interventions that includes social support, knowledge and skills, facilities and services, and menstrual materials and supplies, all of relevance to schoolgirls. UNHCR has a mandate to provide sanitary products for displaced girls (and women), although not specific to the education sector. The *MHM* *in* *Emergencies* *Toolkit,* launched in 2017 with 27 co-publishing organizations, recommends the provision of sanitary products, female-friendly toilets, the provision of MHM and puberty information to girls, and the sensitization of teachers at schools with girls of menstruating age in emergency settings [30]. The WHO hosted a collaborative meeting in 2018 to identify strategic MHM contributions, and possible future guidelines, with a particular focus on the needs of girls, including the intersection with their education. While no global guidelines yet exist, given the varying contexts in which MHM-related programming and policy are implemented, having country-specific guidance may be of greater importance for transforming schools around MHM. However, having global guidance would serve as an important platform from which countries could tailor and adapt guidance at national and local levels.

*Priority* *3:* Advance MHM in schools activities through a comprehensive evidence-based advocacy platform that generates policies, funding, and action across sectors and at all levels of government.

Numerous evidence-based advocacy platforms have emerged that have generated policies, funding and action (see Table 3). Global and national advocacy has served a critical role to advance the MHM in schools’ effort, including coverage by social and mainstream media, and individuals and organizations lobbying for action to address the range of menstruation-related needs of girls. Since 2014, an intensification of efforts focused on reducing menstruation-related stigma and raising awareness about schoolgirls’ MHM experiences and needs. MHM-compendiums like those developed from the 14 country UNICEF, UN Girls’ Education Initiative (UNGEI) and Emory University-led WASH in Schools for Girls (WinS4Girls) effort have provided tools and evidence regarding MHM barriers in school, and subsequently, key messaging for advocacy initiatives (http://wins4girls.org/). Some partnerships have served to create spaces for young people to partake in the MHM conversation. For example, UNICEF and the School of Leadership in Pakistan invited girls and boys to design innovative MHM projects as part of the 16-country Generation Unlimited Youth Challenge. In Nepal and Pakistan, WaterAid used participatory photography so that girls could document experiences and challenges relating to MHM in schools; their exhibitions advocated to families, school authorities and local government for improvements. Although much of the global and local advocacy goes beyond girls in school, such efforts serve to reduce stigma around the topic, and potentially encourage governments and donor institutions to increase resources for MHM in schools specifically. Advocacy efforts are likely to increase pressure on governments and other stakeholders to develop and provide resources to implement relevant policies in education, health or other relevant areas, such as water and sanitation.

**Table 3:** Progress and gaps related to Priority 3

Priority 3: Advance MHM in schools activities through a comprehensive evidence-based advocacy platform that generates policies, funding, and action across sectors and at all levels of government	
Progress	
Inter-national level advocacy	MHM in Schools Virtual conference (Columbia University, UNICEF) (Burgers, Yamakoshi, and Serrano 2019)
	Menstrual Hygiene Day 28th May led by WASH United generates media attention and global momentum, with local, national and regional events organized around the world, including attention to girls in school
	The Global Menstrual Collective (est. 2019) aims to drive and guide improved investment in menstrual health through evidence-based advocacy, including for schools
	African Coalition on MHM has galvanized national advocacy efforts, e.g., Break the Silence in Uganda); other regional initiatives incorporating MHM in schools are South Asian Conference on Sanitation (SACOSAN), and the UNICEF- and GIZ-led WinS-International Learning Exchanges (ILE) (SACOSAN n.d.)
National level advocacy	*Numerous* *advocacy* *campaigns* *are* *led* *by* *national* *alliances,* *international* *organizations,* *and* *UN* *agencies*. *Examples* *include*:
	In Nepal, the Practitioners Alliance on Menstrual Health and Hygiene Management formed in 2016 to bring together different ministries, sectors, researchers, and academics to find solutions to MHM challenges, including in schools
	In India, the MHM advocacy landscape is multi-sectoral and multi-dimensional, with initiatives launched by the private sector such as the Touch the Pickle campaign by Procter and Gamble, WaterAid (the #noshame in menstruation campaign), UNICEF (the #LetsTalkAboutPeriods campaign) and Menstrual Health Alliance India (MHAI), a national inter-agency advocacy group. In Namibia, Power Pad Girls was launched on MH Day 2018 by UNFPA as a 5-day advocacy campaign with MHM messaging. Schoolgirls are a key focus on these advocacy efforts
	*In* *recent* *years,* *the* *MHM* *advocacy* *movement* *has* *expanded* *to* *high-income* *countries,* *although* *sufficient* *empirical* *evidence* *may* *not* *yet* *exist*:
	The U.K., recognizing MHM as an issue of human rights, committed to providing free sanitary products in all primary and secondary schools in early 2020, and developed a period poverty taskforce to focus on tackling stigma, access to products, and data and evidence
	In the U.S., in addition to expanding state efforts to eliminate the tampon tax, some state and city legislators have introduced policies mandating menstrual hygiene products to be provided free of charge in all public schools. In British Columbia and Toronto, Ontario (Canada), the governments passed similar mandates on the provision of products in schools
	Numerous other high-income countries have tackled the tax on sanitary products, although low-income countries led the way on such efforts (e.g. Kenya removed tax in 2004); both have implications for the affordability of products for schoolgirls and female teachers
*Recommendations* *to* *advance* *the* *agenda*	
Effectively translate and disseminate evidence-based research on MHM in schools to varied audiences	
Continue expansion of advocacy efforts to local, rural, and underserved communities	
Engage with growing platforms, alliances, and networks to achieve wider dissemination of messaging around MHM in schools, and clarify how differing collaborations interact, and how gaps between alliances can be filled while minimizing duplications	
Acquire costing information (e.g. cost effectiveness evaluations) of school-based MHM interventions on which to base advocacy	
Integrate MHM into other school-related advocacy campaigns, such as female genital mutilation, reducing child marriage, iron supplementation and HPV vaccination (e.g. distribute girl’s puberty books with HPV vaccines)	

This table comprehensively outlines specific Priority 3 progress and gaps to date

*Priority* *4:* Allocate responsibility to designated government entities for the provision of MHM in schools including adequate budget and monitoring and evaluation [M&E]) and the reporting to global channels and constituents.

A growing number of government entities around the world have shown signs of engagement, responsibility, and ownership of MHM support in schools (see Table 4). Much of this can be attributed to the consistent advocacy efforts and systematic approaches incorporated, for example, by UNICEF’s WinS4Girls programme, which included a clear commitment from a government partner as criteria for selecting countries. In countries such as Kenya, important partnerships between government entities, researchers, and NGOs have played a key role in developing guidelines and uptake of MHM in schools (see Box 2). This provides an important example of research evidence translating into policy development processes at the country level. In many places, ownership and engagement by government actors provide an important mechanism for reducing menstruation-related stigma-related restrictions that girls may face, which in turn may impact girls’ ability to engage productively in school. In Afghanistan, the First Lady and two male ministers spoke openly about the topic of menstruation at a large event on MH Day in 2017; the First Lady of Kyrgyzstan made a statement at a WinS4Girls event; in Nepal, the President recently noted in a speech to Parliament that sanitary pads would be made available free of cost in all community schools; and in India, the subnational Government of Punjab committed to scale-up MHM initiatives in 54,000 schools. It will be important for governments to adopt monitoring and evaluation plans, and for those announcing national or provincial free or subsidized sanitary pad distribution programmes to create adequate disposal mechanisms in schools. As previously highlighted, the Kenya and Philippines policies provide useful examples of the way in which sustained government commitment and committed resources are needed to truly advance progress in schools (see Boxes 2 and 3).

**Table 4:** Progress and gaps related to priority 4

Priority 4: Allocate responsibility to designated government entities for the provision of MHM in schools including adequate budget and monitoring and evaluation [M&E] and the reporting to global channels and constituents	
Progress	
Engagement of multiple ministries on MHM	*Examples* *of* *government* *initiatives* *including* *MHM* *programmes:*
	In Nepal, the ministries of Health, Education, Women and Children, and Water Supply and Sanitation co-signed a declaration in 2018 to make all schools in Nepal menstruation-friendly. MHM-related indicators around girls’ and adolescents’ school absenteeism, female-friendly WASH facilities, and school awareness sessions were integrated into the Ministries of Health and Education plans
	In Cameroon, two national plans formulated MHM goals, including the *National* *Strategy* *for* *the* *Promotion* *of* *Drinking* *Water* *Supplies,* *Hygiene* *and* *Sanitation* *in* *Schools* that focuses on MHM provision to primary schools in priority education zones
	In Punjab, Pakistan the governments demonstrated budgetary commitment; promising to allocate financial resources to implement the Girl Friendly Toilet model into its 2018–2023 strategy, in relation to the education system
	*Multiple* *national* *and* *subnational* *governments* *have* *put* *forth* *MHM* *guidelines* *with* *proposed* *indicators* *for* *measuring* *progress* *since* *2014:*
	Range from MHM in schools operational guidelines to guidelines in other sectors that include MHM-sensitive indicators, or looser guidance documents (e.g. in the Solomon Islands)
	The Ugandan Ministry of Education published WASH guidelines for schools with integrated MHM components, and is now developing national guidelines specific to MHM in schools. The government also published a national training manual for teachers and other actors in MHM in schools, and integrated MHM into the sex education framework and the lower secondary curriculum in 2018
	Other illustrative countries with guidelines relating to MHM in schools include Ethiopia, Eritrea, Zimbabwe and Afghanistan
*Monitoring* *MHM* *within* *education* *systems*	*There* *is* *indication* *that* *more* *and* *more* *countries* *are* *collecting* *information* *on* *WASH* *in* *schools* *through* *Education* *Management* *Information* *Systems* *(EMIS)* *and* *other* *monitoring* *efforts,* *though* *MHM* *indicators* *are* *not* *collected* *uniformly* *within* *or* *across* *countries* *to* *enable* *comparison,* *and* *are* *still* *lacking* *overall*:
	Philippines example (see Box 3)
	MHM indicators have been included in the education management information systems (EMIS) in Zambia and Sindh province (Pakistan)
	Ten MHM-specific indicators were integrated into the school monitoring system in several pilot schools in Sri Lanka, and Rajasthan (India) began collecting state-level data on MHM
	*Despite* *progress,* *in* *many* *countries* *there* *is* *little* *to* *no* *focus* *on* *MHM* *in* *education* *sector* *plans* *and* *limited* *WASH* *indicators* *for* *assessing* *achievements* *and* *progress* *in* *MHM* *in* *schools*
*Recommendations* *to* *advance* *the* *agenda*	
Education sector plans and education policies should include specific attention to MHM and/or its proxies (such as gender segregated toilets). Attention to MHM within policies is needed to assure financial allocations are to address MHM in education systems	
Addressing MHM at the Education sector should be an important requirement to be considered if we want girls to achieve different education outcomes, and it is key to attain Education SDG4	

This table comprehensively outlines specific Priority 4 progress and gaps to date

This priority serves as a critical example of one in which commitment and resources are needed to truly advance progress, with important acknowledgement that there is not a “one size fits all” approach for each country per the responsible entities within a given government. Important to note is that many of the examples identified of government entities engaging on MHM were grounded in quality formative research conducted within each country around the MHM needs of girls in school, and to a lesser extent, intervention research; the latter is essential for assuring an effective and efficient use of limited resources.

*Priority* *5:* Integrate MHM and the capacity and resources to deliver inclusive MHM into the education system.

MHM is beginning to be integrated into country-level education systems, however capacity and resources continue to lag, particularly around incorporating more inclusive approaches (see Table 5). In addition, limited national policies currently exist that explicitly incorporate attention to menstruation, which hinders the allocation of budget line items to build capacity and deliver effective programming. Yet a number of governments are taking promising steps towards increasing responsibility for providing MHM in schools. For example, in Zambia, the Ministry of General Education is rolling out the country’s MHM programme. In most countries, this has not yet translated to national action that is inclusive and accompanied with implementation and monitoring resources. While policies, standards and guidelines are growing, clear responsibility for implementation is not always clearly articulated, with financing often insufficient. The use, integration, operationalization, and scale-up of guidelines, for example, remains limited, even where there are plans to do so. In a number of countries, activities have been initiated. For instance, Nepal’s *Integrating* *Menstrual* *Health* *and* *Hygiene* *in* *School* *Education,* developed by the Family Welfare Division, is being piloted in different districts, supported by the German Corporation for International Cooperation GmbH (GIZ). Indonesia has used the National Health Programme (UKS) to test a roll out of an MHM package in schools, supported by the Ministries of Education and Health. Madagascar is planning a scale-up of MHM school activities in 2019, although smaller scale, and to date, Somalia has only implemented its institutional sanitation and hygiene promotion in a fraction of schools. The Gambia began to roll out a pilot initiative to implement minimum guidelines in 2018. In 2017, a study found that less than 20% of schools in rural Ethiopia, Kenya, Mozambique, Rwanda, Uganda and Zambia had four out of the five recommended menstrual hygiene services (e.g. sex-separate facilities, water supply, door with lock, waste disposal bin) [31]. And in high-income countries, legislative attention to addressing MHM in schools only very recently emerged. Much progress is needed across this priority to reach the vision of 2024, although the examples provided are useful for highlighting efforts underway, and potential models for other countries to follow. Truly impactful change on addressing the menstruation-related needs of girls and female teachers will only occur when policies—national or sub-national—are embedded within government institutions with the needed capacity and resources to deliver programming, and monitor impact and outcomes.

**Table 5:** Progress and gaps related to priority 5

Priority 5: Integrate MHM and the capacity and resources to deliver inclusive MHM into the education system	
Progress	
Resources and technical support for governments	*In* *many* *contexts,* *multi-sectoral* *actors* *are* *contributing* *to* *the* *delivery* *of* *MHM* *within* *the* *education* *system:*
	An example includes USAID’s support for renovations of government school WASH facilities delivered by Plan India and Save the Children Malawi
	*Multiple* *social* *enterprises* *provide* *menstrual* *products* *and* *MHM* *education* *to* *schoolgirls:*
	This illustratively includes Afripads in Uganda and ZanaAfrica in Kenya; Mariam Seba Sanitary Products factory in Ethiopia, and numerous menstrual cup companies (https://menstrualcupcoalition.org/)
	Product-based initiatives have developed new innovative products for girls to expand their choices, and some provide better solutions for the environment
	*Education* *initiatives* *have* *generated* *useful* *approaches* *and* *materials* *using* *child-centered* *design*
	Some have focused on particular marginalized groups including girls with disabilities, girls from minority ethnic groups, and targeting boys with information about menstruation
	A few countries are integrating MHM into the national curriculum
	*Philanthropic* *organizations* *have* *partnered* *with* *NGOs* *and* *national* *governments* *to* *implement* *promising* *initiatives* *towards* *MHM* *school* *integration*
	Dubai Cares funded Sesame Workshop and World Vision to scale up the WASH UP! Girl Talk programme to 150–200 schools in rural Zimbabwe. The approach includes delivery of an educational curriculum on health, puberty, WASH, and MHM to be implemented by public school teachers, with monitoring conducted during the scale-up
	*Other* *innovative* *activities* *that* *have* *been* *implemented* *to* *improve* *MHM* *in* *schools* *include:*
Integrating more inclusive MHM	School clubs, peer-to-peer education, puberty books, online apps and games to promote knowledge and hygiene practices related to MHM, and sewing sanitary pads activities by both boys and girls in school
	*Some* *national* *governments* *have* *dedicated* *resources* *to* *providing* *more* *inclusive* *approaches* *to* *MHM,* *in* *some* *countries,* *NGOs* *have* *partnered* *with* *governments* *to* *deliver* *such* *service:*
	In Kenya, Huru International in collaboration with the Ministry of Health and local Ministry of Education offices has delivered an MHM programme for girls with disabilities
	In Uganda, the PEPFAR-funded Determined Resilient Empowered AIDS-Free Mentored and Safe (DREAMS) programme has included boys in addressing MHM in schools
	In Chad, UNICEF supported WASH in schools programming included attention to nomadic herders, and children in refugee settings in their MHM efforts
	In India, WaterAid and UNICEF designed and delivered MHM in schools for residential schools in one state that are specifically for students from minority tribal and ethnic groups
*Recommendations* *to* *advance* *the* *agenda*	
Increased inclusiveness, including attention to the needs of transgender youth and other vulnerable populations	
Sustainability of local supply chains is still to be demonstrated in many locations, particularly when menstrual hygiene supplies are subsidized	
Address continued stigma around menstruation that serves as a barrier to MHM integration into education systems even where there is government commitment and engagement	
Attention is needed to institutionalize MHM information to ensure that it is delivered at scale through the education system, and that teachers have the confidence and capacities to teach the topic	
*National* *governments* *must* *lead* *the* *coordination* *of* *external* *inputs,* *combined* *with* *domestic* *resources* *and* *commitment* *to* *MHM,* *in* *order* *to* *achieve* *scale* *across* *a* *country*	

This table comprehensively outlines specific Priority 5 progress and gaps to date

### Expansion beyond schools

Although not within the scope of what could be included in this paper, there has been important progress made beyond schools in relation to menstruation, such as the inclusion of questions on menstruation and menarche in national levels surveys (MICS and DHS) as noted above, and the WHO’s 2018 Guidelines on Sanitation and Health which emphasize the importance of MHM-appropriate facilities, representing useful global guidance even if not specific to schools [32]. Additional efforts are underway to understand and address the needs of out of school girls, women, those in the workplace, and a more diverse range of menstrual experiences among populations in varying contexts. Part of this effort includes the evolution of definitions and terminology, such as Menstrual Health and Hygiene, defined well in UNICEF’s guidance [33], and a new definition for Menstrual Health is being developed by members of the Global Menstrual Collective There is also a critical need to assess and monitor how COVID-19 may widen disparities in relation to menstruation. The loss of family livelihoods may further exacerbate gendered access to and engagement in education. Menstrual health and hygiene should be considered as part of a safe return to school that considers all the gendered needs of girls.

### Research and policy recommendations for advancing the agenda

To truly advance the MHM in Ten agenda in countries around the world, rigorous implementation research is needed to enable governments and donors to channel resources effectively, including contributing to the design of appropriate policies and related programs in a each country context. As differing government entities may be responsible and/or relevant for transforming girls’ experiences of menstruation in relation to school in each country, such as a sanitation and hygiene policy in one country, an education policy in many countries, and a health or gender related policy in other countries, it is essential that a foundation of relevant evidence be generated and translated as needed. To truly be impactful, enacted policies (or the relevant given entity in a sector, such as *Education* *Sector* *Plans*) should be linked with budget line items, and have operational plans and strategies that incorporate monitoring and evaluation to ensure implementation is happening as intended, and delivers the desired outcomes. As incorporated in this paper, a small number of countries are leading the way through their evidence-informed initial national level policy efforts [34].

### Limitations

This MHM in Ten progress update was conducted through a desk review and outreach to the ever-growing network of experts and organizations working on menstrual health and hygiene, with an expanded number of individuals and organizations contacted. This brief review does not represent all activities worldwide and much of the MHM progress may not be identified easily through existing documentation, as government documents may not be shared widely and/or be available only in local languages. Therefore, this review likely under-represents progress. Nevertheless, we hope that this review will inspire reporting and the expansion of the MHM in Ten network to enable a stronger review by 2024, with gains achieved across the five priorities, either through direct action or indirect efforts that contribute to meeting the vision.

Box 2: The Philippines: monitoring an MHM policy in the education system *The* *Policy:* In 2016, the Philippines Department of Education (DepEd) passed the Comprehensive Policy and Guidelines on Water, Sanitation and Hygiene (WASH) in Schools (DepEd Order No. 10). The policy aims to “improve hygiene and sanitation practices among the learners to enable them to develop life-long positive hygiene and sanitation behaviors,” and decrees that “a system and support mechanisms for effective menstrual hygiene management shall be ensured in all schools.”

The DepED WASH in Schools technical working group crafted implementation mechanisms, including the National Three-Star On-line Monitoring System (OMS), Recognition, and Incentive System. WASH indicators were incorporated into the current Enhanced Basic Education Information System (E-BEIS) including indicators on WASH in schools operation and maintenance, and access to MHM supplies.

*Monitoring*: The OMS and E-BEIS serve as monitoring, reporting and planning tools at local, sub-national and national levels. The following MHM related data are being monitored and reported:

- *regular* *supply* *of* *soap* *and* *water* *in* *individual* *handwashing* *stations* *near* *toilets;*
- *disposal* *of* *MHM* *materials;*
- *private,* *secure,* *functional,* *gender* *segregated* *toilets;*
- *resting* *space* *for* *MHM;*
- *availability* *of* *MHM* *related* *IEC* *materials*.

This monitoring has allowed the Philippines government to promote awareness of school administrators on their own Schools WASH and MHM status and provided a platform by which administrators are guided to maximize resources, and continuously improve their star status and reach national standards.

*Learning* *Points:*

- This approach enables supervisors at sub-national levels to monitor implementation of the program and provide specific targets for technical assistance to ensure quality implementation and the incorporation of key MHM core messages to augment what is being taught in the curriculum.
- After three years of implementation, awareness on WASH and MHM has improved, there has been increased support for construction of gender segregated toilets, and girls’ toilets are now better designed to allow for a bidet or a washing area, and the provision of covered garbage bins for used napkins.
- Visible MHM information and adolescent health development information are posted on toilets, school health boards, and in hygiene areas, and MHM messages are given emphasis in curriculum instruction, particularly in homeroom guidance, science, social science, and health classes.
- A key enabler for progress included using the WASH in schools program as an entry point for addressing MHM, while key barriers have included on-going menstrual stigma and lack of prioritization by the Education sector.

Box 3: Kenya: expanding policy and implementation of MHM in the education system *The* *Policy:* The Ministries of Health and of Education reviewed the National School Health Policy. The revised policy has strong statements on MHM and asserts that “MHM is a crucial element of the School Health Policy, being important for dignity, gender equality and the human rights of women and girls. This policy recognizes that women and girls who experience challenges with MHM will also experience negative effects on multiple areas of life; relevant to the human rights of women and girls, including in particular the rights to health, work and education, as well as gender equality”. The school health policy outlines clear action points, indicators and objectives to achieve comprehensive MHM across schools in Kenya. Kenya has also developed an MHM Policy, and a strategy to provide direction on how to implement MHM activities in Kenya and to promote integration of MHM and collaboration of all sector actors is also underway.

In 2017 the President of Kenya signed the Basic Education Amendment Act No. 17 of 2017 which states that the government shall “provide free sufficient and quality sanitary towels to every girl child registered and enrolled in a public basic education institution who has reached puberty and provide a safe and environmentally sound mechanism for disposal of the sanitary towels.”

*Implementation:* Since 2017, Kenya has reviewed the education system in order to adopt a competency-based curriculum. The Ministry of Education, Ministry of Health and MHM stakeholders in Kenya developed the ’Menstrual Hygiene Management Teachers’ Handbook’ as a reference book for training and delivering MHM sessions to learners in primary schools. The Handbook is under review by the Kenya Institute of Curriculum Development so that it can be used to train teachers. In addition, the Ministry of Health, Ministry of Education and Ministry of Water and Sanitation have reviewed the ‘Standards for WASH infrastructure in schools’ and incorporated a strong component of MHM. This book provides guidance on how to construct WASH friendly facilities in schools, and in particular female friendly toilets. The book includes architectural plans, bill of quantities, schedules of work and WASH requirements for toilets that meet standards as MHM friendly.

The government, through the State Department of Gender at the Ministry of Public Service, Youth and Gender Affairs has been distributing free sanitary pads to targeted girls in public primary schools. The Ministry of Education has a vote head that is used for among other things, the purchase of emergency sanitary pads for school girls for when the school has not received their consignment of sanitary pads. The funds are sent to the headteachers of the various schools. In addition, the Ministry of Education in Kenya is reviewing the National Education Management Information System to include MHM indicators, and has allocated funds to public schools for school infrastructure development. These have been used to construct WASH facilities in schools.

*Challenges:* Funding for the implementation of the various guidelines and policies remains a challenge, and the lack of clear indicators against which to measure progress hampers the collection of data to assess implementation. Collaboration between various relevant ministries remains a challenge, although the Ministry of Health continues to invite other ministerial engagement (e.g. Gender, Environment and Water).

## Conclusions

MHM is now recognized globally as a definitive public health and development issue, with substantial increase in financial and human capital committed toward this topic. While progress has been made across the five priorities in a range of countries, and is moving towards the vision for 2024 directly or indirectly, much remains to be done. Gaps in progress cannot be filled until resources and political commitments are made to transform schools for menstruating girls. The SDGs present an opportunity for greater prioritization of the issue of MHM for schoolgirls, including increased integration across sectors and improved monitoring of progress. They also provide pressure on governments to enact relevant policies that enable transforming education systems at scale. Progress was primarily identified in countries across Priorities 1, 2 and 3, with Priorities 4 and 5, requiring government engagement and capacity, highlighted as areas for attention in the second half of the ten-year agenda.

Key recommendations for actions that will advance the agenda include support for learning from implementation of government programmes and policies to share across country governments, longitudinal research to measure relevant impact and outcomes; improved investment in the evidence base for addressing MHM in schools, particularly research targeting the most underserved; a better understanding of costs and effectiveness, and the benefits of comprehensive, cross-sectoral approaches. Together these actions will contribute to meeting the vision that all girls in 2024 are knowledgeable and comfortable with their menstruation, and able to manage their menses in school in a comfortable, safe and dignified way.

## Data Availability

Not applicable.
